# Co_9_S_8_ nanoparticle-decorated carbon nanofibers as high-performance supercapacitor electrodes†

**DOI:** 10.1039/c8ra04296a

**Published:** 2018-08-03

**Authors:** Ning Zhang, Wencong Wang, Changqing Teng, Zongxiao Wu, Ziran Ye, Mingjia Zhi, Zhanglian Hong

**Affiliations:** State Key Laboratory of Silicon Materials, School of Materials Science and Engineering, Zhejiang University 38 Zheda Road Hangzhou 310027 China; Department of Applied Physics, Zhejiang University of Technology Zhaohui Campus Hangzhou 310014 China

## Abstract

This work reported Co_9_S_8_ nanoparticle-decorated carbon nanofibers (CNF) as a supercapacitor electrode. By using a mild ion-exchange method, the cobalt oxide-based precursor nanoparticles were transformed to Co_9_S_8_ nanoparticles in a microwave hydrothermal process, and these nanoparticles were decorated onto a carbon nanofiber backbone. The composition of the nanofibers can be readily tuned by varying the Co acetate content in the precursor. The porous carbon nanofibers offered a fast electron transfer pathway while the well dispersed Co_9_S_8_ nanoparticles acted as the redox center for energy storage. As a result, high specific capacitance of 718 F g^−1^ at 1 A g^−1^ can be achieved with optimized Co_9_S_8_ loading. The assembled asymmetric supercapacitor with Co_9_S_8_/CNF as the cathode showed a high energy density of 23.8 W h kg^−1^ at a power density of 0.75 kW kg^−1^ and good cycling stability (16.9% loss over 10 000 cycles).

## Abstract 2

Through electrospinning and the ion-exchange method, Co_9_S_8_ nanoparticle-decorated carbon nanofibers (Co_9_S_8_/CNF) have been fabricated, and exhibit good supercapacitor performance.

## Graphical abstract

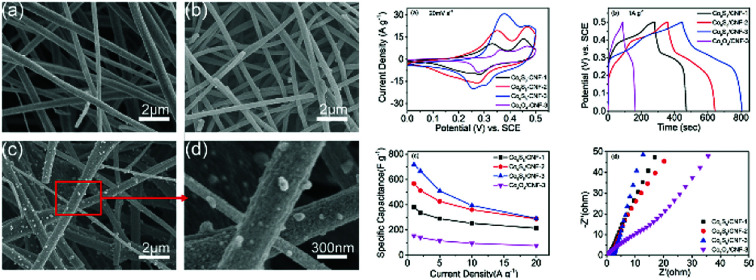

## Introduction

In recent years, metal sulfides have attracted lots of interest for energy storage devices,^1–8^ since they have a richer surface redox capability compared with their oxide counterparts.^9,10^ This ensures metal sulfides have higher activities in energy storage and conversion reactions. Among the various metal sulfides, Co_9_S_8_ has proved itself as a promising material for supercapacitor electrode applications due to its high theoretical capacitance.^11–13^ Different types of cobalt sulfide nanostructures have been explored as supercapacitor electrodes, including nanospheres,^14,15^ nanocages,^16^ nanoflowers,^17–19^ and so on.^20,21^ Extraordinarily high specific capacitance of over 1600 F g^−1^ has also been reported in vapor grown Co_9_S_8_ films.^12^

However, similar to metal oxides, metal sulfides suffer from possible aggregation and poor electrical conductivity, which prevents full utilization of their capacitance.^22^ It is then reasonable to incorporate Co_9_S_8_ phases into carbon nanostructures to construct the composite, in which the carbon acted as the support to offer higher electronic conductivity and porous structure, while the better utilization of the Co_9_S_8_ can be accomplished.^23,24^ For instance, the CoS_*x*_/carbon core–shell nanospheres were decorated on electrospun carbon nanofibers (CNF), and specific capacitance of 497 F g^−1^ at 0.5 A g^−1^ has been reported.^25^ The enhanced performance was attributed to the synergistic effect from the enhanced conductivity and high specific capacitance of CoS_*x*_. Co_9_S_8_ nanoflakes/graphene composite was also prepared. Due to the better conductivity of graphene phase, high specific capacitance of 808 F g^−1^ at 5 mV s^−1^ was achieved, which was more than twice to that of pristine Co_9_S_8_.^13^

In this work, fine Co_9_S_8_ nanoparticles were decorated on electrospun carbon nanofibers to form the Co_9_S_8_/CNF composite nanofibers. Such Co_9_S_8_ nanoparticles were obtained by mild ion-exchange synthesis route and were uniformly dispersed in CNF, which acted as the porous conductive matrix. This microstructure promoted both the electronic and ionic conductivity of the electrode and maintained the intrinsic activity from metal sulfide. As a result, high specific capacitance (as high as 718 F g^−1^ for the composite) has been obtained. The assembled asymmetric capacitor also showed high energy density and good cycle stability (83.1% remained over 10 000 cycles).

## Experimental

### Materials synthesis

The cobalt acetate/PAN (polyacrylonitrile) precursor composite nanofibers were prepared by electrospinning method in the first step. The electrospinning solution was prepared as following. Briefly, 0.8 g of PAN (MW ≈ 150 000) was dissolved in 10 mL of *N*,*N*-dimethylformamide (DMF) under stirring.^26^ After that, different amounts of cobalt acetates were added into the above solution. The concentrations of Co^2+^ were set to 0.125 M, 0.25 M and 0.5 M, respectively. The mixture was then stirred at 120 °C for 1 h until a red and sticky solution was obtained. The above solution was then loaded into plastic syringe and electrospinning experiment was conducted. A high voltage of 18 kV was applied between the needle and the sample collector (distance 15 cm). The typical feeding rate was 1 mL h^−1^.

The obtained cobalt acetate/PAN composite nanofibers were then dried at 80 °C for 12 h in oven, and then stabilized in air at 250 °C for 1 h with a heating rate 2 °C min^−1^. The stabilized nanofibers were then immersed into 0.1 M thioacetamide/H_2_O solution and sulfurized *via* a microwave hydrothermal method. The temperature and the duration were 120 °C and 1 h. During the microwave hydrothermal treatment, the cobalt oxide-based phase was transferred to sulfide phase. The sulfurized nanofibers were finally heated at 600 °C for 3 h under nitrogen to give Co_9_S_8_/CNF samples. The composite nanofibers were denoted as Co_9_S_8_/CNF-1, Co_9_S_8_/CNF-2, Co_9_S_8_/CNF-3 based on the concentration of Co^2+^ (0.125, 0.25 and 0.5 M). In addition, control experiment was conducted to prepare Co_3_O_4_/CNF-3 composite without sulfurization process.

### Characterization

The morphology of the samples was observed by scanning electron microscope (SEM, Hitachi-S4800) and transmission electron microscope (TEM, JEM1200EX). The crystalline phase analysis was performed by X-ray diffraction (XRD-6000, Shimadzu) using Cu Kα radiation (*λ* = 0.15 nm). Thermogravimetric analysis (TGA, TA-Q500) was carried out to estimate the content of Co_9_S_8_ in the prepared composite. The pore size distribution and specific surface area were calculated according to N_2_ adsorption–desorption method using Autosorb (Quantachrome Instrument Corp). The Fourier Transform infrared spectroscopy (FTIR) was performed on Nicolet 5700 spectrophotometer. The X-ray Photoelectron Spectroscopy (XPS) characterization was performed on Escalab250Xi instrument.

All the electrochemical measurements were carried out on an electrochemical workstation (CHI660E, ShangHai ChenHua Co., Ltd, China) at room temperature. The supercapacitor tests were performed in both three-electrode and two-electrode configurations in 6 M KOH electrolyte. The working electrode was prepared by mixing 80 wt% of active materials (Co_9_S_8_/CNF), 10 wt% of Ketjen black and 10 wt% polyvinylidene fluoride (PVDF) dissolved in *N*-methyl pyrrolidone (NMP). The slurry was subsequently casted onto Ni foam and dried at 60 °C in oven overnight. In the three-electrode system, Pt and Saturated Calomel Electrode (SCE) was used as the counter and reference electrode, respectively. In the two-electrode system, the asymmetric supercapacitors were built using the as-prepared working electrode as positive electrode and active carbon nanofibers (ACNF) as negative electrode.

The electrochemical performances of the supercapacitors were evaluated by characterizing their cyclic voltammetry (CV), galvanostatic charge/discharge (GCD) and electrochemical impedance spectroscopy (EIS). The voltage window was from 0 to 0.5 V *vs.* SCE for the three-electrode system, and 0 to 1.5 V for the asymmetric Co_9_S_8_/CNF-ACNF cell. The GCD were tested at current density ranging from 0.5 A g^−1^ to 20 A g^−1^ and CV were tested at scanning rate of 2 mV s^−1^ to 200 mV s^−1^. The EIS was measured at open circuit with 30 mV AC potential in the frequency ranged from 100 kHz to 0.01 Hz. Cycle stability measurements were tested at 10 A g^−1^ current density for 10 000 cycles.

The specific capacitance (*C*) of the device was calculated based on the integration of the discharge curve as following1
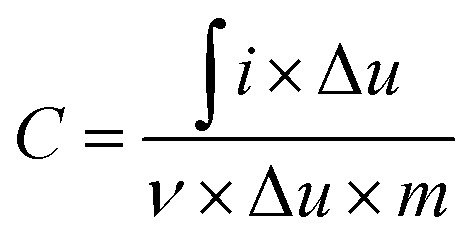


The energy density (*E*) and power density (*P*) were calculated according to the following equation:2
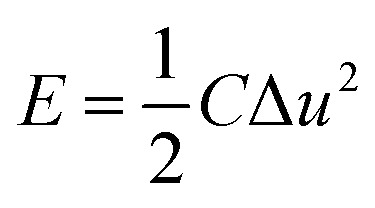
3
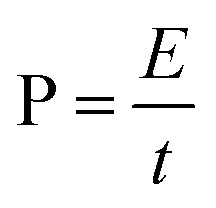
where Δ*u* is the potential, *v* is the potential scan rate (mV s^−1^), *i* is the discharge current (A), *t* is the discharge time (s), and *m* is the mass (g) of the active materials on the electrode.

## Results and discussion

**Fig. 1:**
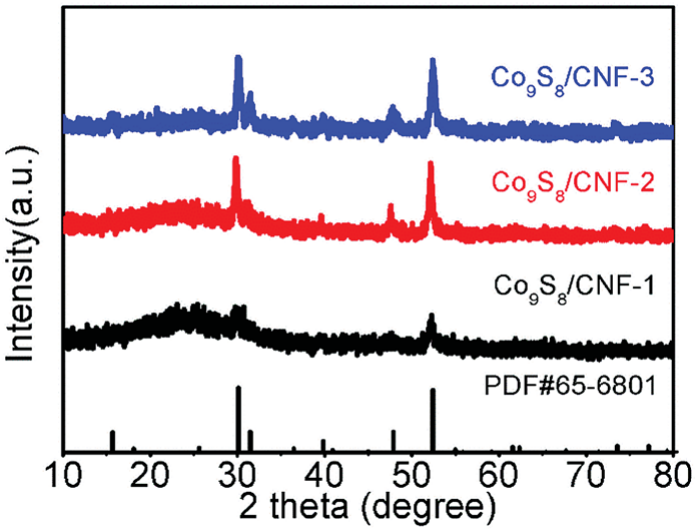
XRD pattern of the Co_9_S_8_/CNF samples.

Fig. 1 shows the XRD patterns of the Co_9_S_8_/CNF series samples. From the patterns, one can conclude that Co_9_S_8_ phase can be successfully formed after the microwave-assisted sulfurization and carbonization process, since the diffraction peaks can be identified as standard Co_9_S_8_ (JCPDF65-6801) in the patterns. The peak intensity in Co_9_S_8_/CNF-1 is rather weak, which may be due to that the content of the sulfide phase was not high. However, in Co_9_S_8_/CNF-2 and Co_9_S_8_/CNF-3 samples, clear diffraction peaks can be observed, which indicates the Co_9_S_8_ in the composite nanofibers had good crystallinity. In contrast, the XRD pattern of Co_3_O_4_/CNF-3 sample matched with Co_3_O_4_ phase as shown in Fig. S1.† From the above comparison, one may propose the formation mechanism of Co_9_S_8_ phase in the composite nanofibers. During the stabilization process, the cobalt acetate was decomposed into cobalt oxide by heating. The afterward microwave hydrothermal step initiated the ion-exchange reaction to transform Co_3_O_4_ into Co_9_S_8_, since TAA can decompose and release sulfur contained reactants.^3,27–31^ It is also known that the material with lower solubility (*K*_sp_) is more thermodynamically stable than that with higher *K*_sp_ value. The ionic radius of S^2−^ (0.18 nm) anions is also close to the oxygen (0.14 nm) anions. Thus, Co_9_S_8_ nanoparticles were formed. The high temperatures in the carbonization process helped to improve the crystallinity of the cobalt sulfide and Co_9_S_8_ nanoparticles can form.

**Fig. 2:**
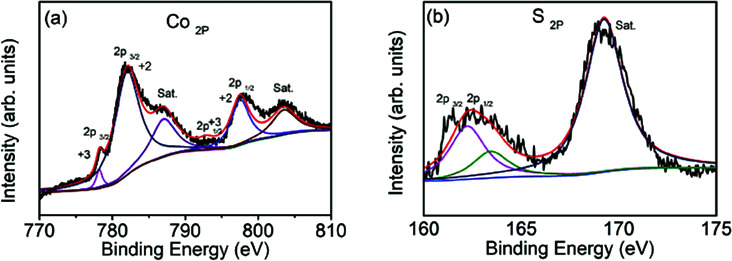
XPS spectra of Co_9_S_8_/CNF-3, (a) Co 2p core level, and (b) S2p core level.

More details about the elemental composition of the Co_9_S_8_/CNF samples were investigated by XPS. Fig. 2 gives the core level scans of Co 2p and S 2p of the Co_9_S_8_/CNF-3 sample. The high resolution Co 2p XPS spectrum (Fig. 2(a)) shows the presence of Co^2+^ and Co^3+^. The peaks at 778.2 eV and 792.9 eV were belonged to Co^3+^ 2p_3/2_ and 2p_1/2_, and the two peaks at 782.0 eV and 797.5 eV were assigned to Co^2+^ 2p_3/2_ and 2p_1/2_, respectively.^32,33^ The S 2p spectrum can be divided into two main peaks and one satellite peak, as shown in Fig. 2(b). The two peaks at 161.4 eV and 162.6 eV can be attributed to the metal–sulfur bonds.^34,35^ These XPS results are consistent with the XRD analysis mentioned above and suggest that Co_9_S_8_ is successfully formed. The surface composition of the composite nanofibers was also estimated from the XPS full spectra and the data was shown in Table S1.† It can be seen that the content of the Co_9_S_8_ in the samples increased as the Co^2+^ ions concentration increased in the precursor.

The formation of Co_9_S_8_ was further confirmed by FTIR spectroscopy. Fig. S2† gave the FTIR spectra taken from the Co_9_S_8_/CNF samples. The bands located at 606 and 470 cm^−1^ were assigned to Co_9_S_8_ phase, which has been discovered in metal sulfide materials.^3,36^

The content of Co_9_S_8_ in the composite nanofibers were examined by TGA method, and the result was shown in Fig. S3.† The TGA of the pristine Co_9_S_8_ was also presented in Fig. S4† as the reference. The samples were heated from 50 °C to 800 °C in air. There were three stages of weight loss in the Co_9_S_8_/CNF samples during the heating process. At first (before 250 °C), there was a slight weight loss which probably was attributed to the evaporation of water remained in the sample. When the temperature raised from 250 °C to 550 °C, two competition process co-existed. On one side, because of the partial oxidation of Co_9_S_8_,^37^ the weight of the sample will be slightly increased which can be seen from the pristine Co_9_S_8_ in Fig. S4(b).† On the other side, the decomposition of carbon will cause a huge weight loss. Considering these two process, the composite nanofibers showed weight loss. At the last step, the cobalt sulfide converted into cobalt oxide when the temperature was above 700 °C and the weight became constant. The final remained weight of the three samples was 18.9%, 28.5% and 38.8% as shown in Fig. S3,† and from Fig. S4(b)† it is known that the weight loss was about 11.8% when the pristine Co_9_S_8_ converted to Co_3_O_4_.^38^ Thus, the Co_9_S_8_ contents in Co_9_S_8_/CNF-1, Co_9_S_8_/CNF-2, and Co_9_S_8_/CNF-3 samples can be estimated to be ∼21.4%, 32.3% and 44.0%, respectively.

**Fig. 3:**
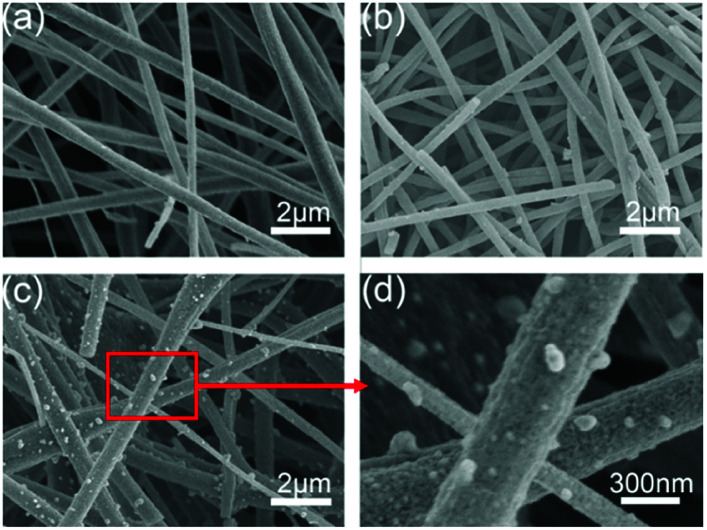
SEM images of the Co_9_S_8_/CNF samples, (a) Co_9_S_8_/CNF-1, (b) Co_9_S_8_/CNF-2, (c) and (d) Co_9_S_8_/CNF-3.

The morphology of the Co_9_S_8_/CNF was characterized by SEM. Fig. 3 show the corresponding images for the samples. From Fig. 3(a) (Co_9_S_8_/CNF-1, with the lowest Co_9_S_8_ loading), it can be seen that the sample was composed of stacked nanofibers, which is typically seen in the electrospun samples. The diameter of an individual fiber was ∼300 nm and the length can reach to tens of microns. The stacked nanofibers formed a porous matrix, which is beneficial for continuous electron transfer and ion penetration.^39^ When the Co_9_S_8_ content increased, the porosity of the fiber network can be well retained as shown in Fig. 3(b). In Fig. 3(c) and (d) (which is the magnification of Fig. 3(c)), when the Co_9_S_8_ content was the highest, numerous nanoparticles were found to decorated on the fiber surface, and the size was estimated to be 100 nm. A comparison of the Co_3_O_4_/CNF-3 can be found in Fig. S5(a),† which revealed the similar porous structure. In Fig. S5(b),† it can be seen that the surface of the fiber was coarse, and small Co_3_O_4_ can be identified. The above morphology analysis shows that the Co_9_S_8_ nanoparticles prepared by ion-exchange method possessed the similar morphology as their oxide counterpart.

**Fig. 4:**
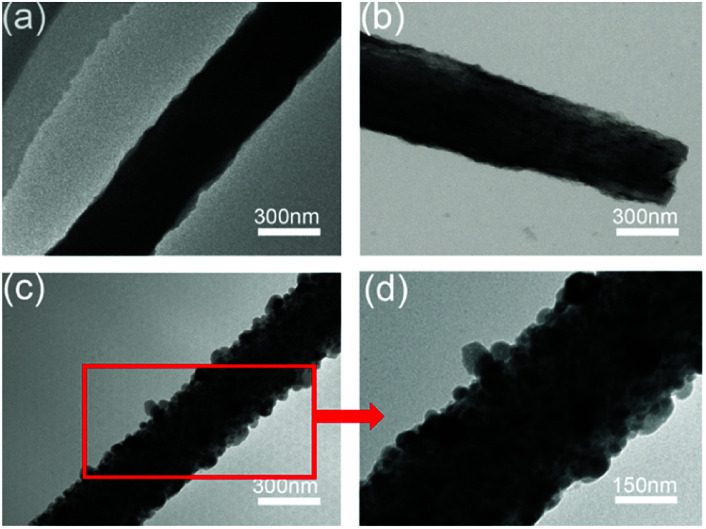
TEM images of the Co_9_S_8_/CNF samples, (a) Co_9_S_8_/CNF-1, (b) Co_9_S_8_/CNF-2, (c) and (d) Co_9_S_8_/CNF-3.

The microstructure of the Co_9_S_8_/CNF samples was further characterized by TEM and showed the similar trends. Fig. 4(a) shows the TEM image taken from Co_9_S_8_/CNF-1. Fibers with coarse surface can be observed, and the Co_9_S_8_ phase can be barely identified since its content was low. In Fig. 4(b) (Co_9_S_8_/CNF-2), fine nanoparticles with darker contrast can be clearly seen. In Fig. 4(c) and (d) (Co_9_S_8_/CNF-3), one may see a large number of Co_9_S_8_ nanoparticles with the size of ∼100 nm appeared on the surface of the carbon nanofibers, which agreed well with the SEM characterization result. These nanoparticles were firmly attached on the CNF surface, and such structure prevented the aggregation of the nanoparticles. The above morphology evolution indicated that the existence of the Co_9_S_8_ nanoparticles was more evident and the size became larger when the Co_9_S_8_ phase increased.

**Fig. 5:**
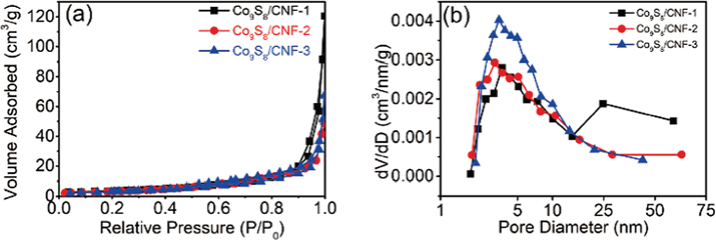
(a) N_2_ adsorption/desorption isothermal curves for Co_9_S_8_/CNF samples, and (b) the corresponding pore size distributions calculated from the adsorption branch.

Fig. 5(a) shows the N_2_ adsorption/desorption isothermal curves for the samples. The isotherm showed typical type IV behavior, indicating the presence of the mesopores. In addition, large N_2_ uptake can be found when the relative pressure was larger than 0.9, which is the sign of the macropores. The existence of both mesopores and the macropores are beneficial for high performance supercapacitor electrode, since they can act as the ion transfer pathway and ion reservoir, respectively.^23^ The calculated surface area for Co_9_S_8_/CNF-1, Co_9_S_8_/CNF-2, and Co_9_S_8_/CNF-3 samples were 13.61 m^2^ g^−1^, 12.20 m^2^ g^−1^ and 12.02 m^2^ g^−1^, respectively. Such values are comparable to the reported CoS_*x*_/C nanosphere decorated carbon nanofibers samples.^25^ The corresponding pore size distribution calculated from the adsorption branch in the mesopore range can be found in Fig. 5(b). A peak at 4 nm can be found in all samples, which may be due to the decomposition of the polymer in the carbonization process, and the Co_9_S_8_/CNF-3 possessed the narrow pore size distribution.

**Fig. 6:**
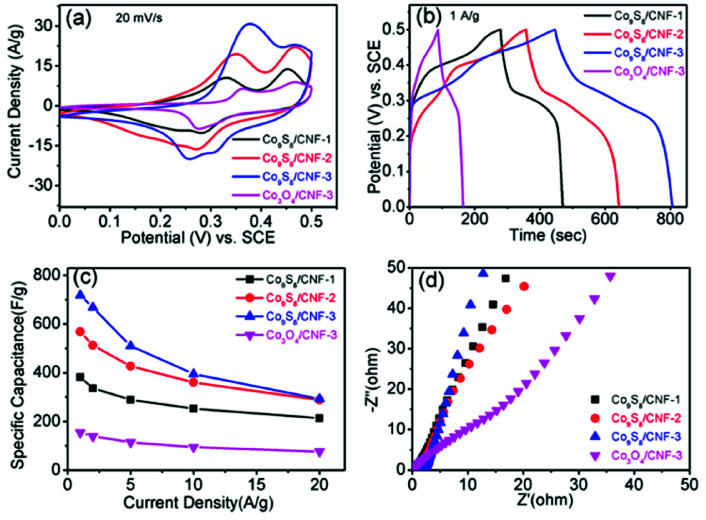
(a) CV curves of the Co_9_S_8_/CNF and Co_3_O_4_/CNF electrodes at 20 mV s^−1^ scan rate, (b) GCD curves of the Co_9_S_8_/CNF and Co_3_O_4_/CNF electrodes at 1 A g^−1^ current density, (c) the he specific capacitance *versus* current densities and (d) the EIS spectra of different electrodes.

The electrochemical performance of the composite nanofibers was firstly evaluated in 3-electrode configurations. Fig. 6(a) shows the corresponding CV curves of the electrodes at the scan rate of 20 mV s^−1^. For comparison, the CV curves of Co_3_O_4_/CNF-3 sample was also demonstrated. From the curves for the Co_9_S_8_/CNF samples, pairs of broad redox peaks can be observed (two oxidation peaks between 0.35 to 0.45 V *vs.* SCE but only one broad reduction peaks between 0.25 to 0.32 V *vs.* SCE),^40,41^ which are attributed to the multiple oxidation steps of Co_9_S_8_ in alkaline electrolyte by the following equations:^41,42^

4 Co_9_S_8_ + OH^−^ = Co_9_S_8_(OH) + e^−^

5 Co_9_S_8_(OH) + OH^−^ = Co_9_S_8_OH + e^−^

Specifically, two well defined reduction peaks can be found in Co_9_S_8_/CNF-3 sample, while one reduction peak was found in other samples. Such phenomenon has been reported in the previous Co_9_S_8_ supercapacitor electrode. This may be attributed to that the Co_9_S_8_/CNF-3 sample possessed the highest Co_9_S_8_ content. The above observation confirms the charge storage in Co_9_S_8_/CNF electrodes is mainly accomplished by the fast-redox reactions in Co_9_S8 phase. The peak current density can be used to evaluate the specific capacitance of the electrode, which followed the order of Co_3_O_4_/CNF-3 < Co_9_S_8_/CNF-1 < Co_9_S_8_/CNF-2 < Co_9_S_8_/CNF-3, and the detail CV curves measured at different scan rates for Co_9_S_8_/CNF-3 can be found in the ESI Fig. S6(a).† This indicated Co_9_S_8_/CNF-3 had the largest specific capacitance, which was proved by the galvanotactic method. Fig. 6(b) demonstrated the constant current charge–discharge curves of the corresponding electrodes at 1 A g^−1^. All the curves had the shape of quasi-triangle with plateaus, which matched with their CV curves in which clear redox peaks were found. The longest discharge time was found in Co_9_S_8_/CNF-3 electrode and decreased in the same order as the CV results. The specific capacitance at 1 A g^−1^ was 718 F g^−1^, 568 F g^−1^ and 382 F g^−1^ for Co_9_S_8_/CNF-3, Co_9_S_8_/CNF-2, and Co_9_S_8_/CNF-1 electrodes, respectively. Such values are much larger than conventional electrical double layer capacitance obtained in pristine porous carbon nanofibers (typically less than 200 F g^−1^) and are superior to the reported CoS_*x*_-CNF composite nanostructures. For examples, CoS_*x*_@carbon core–shell nanospheres decorated carbon nanofibers electrode showed 497 F g^−1^ capacitance at 0.5 A g^−1^ current density.^25^ In addition, the performance of Co_9_S_8_/CNF-3 electrodes was also better than most of the reported pristine cobalt sulfide nanostructures, such as hollow Co_9_S_8_ nanospheres^14^ (306 F g^−1^ at 0.1 A g^−1^), cobalt sulfide nanotubes^43^ (285 F g^−1^ at 0.5 A g^−1^), flower-like cobalt sulfide hierarchitectures^17^ (674 F g^−1^ at 3 A g^−1^), and 3D flower-like Co_9_S_8_ hierarchical architectures^19^ (522 F g^−1^ at 0.5 A g^−1^). Considering the Co_9_S_8_ content was 44% in the composite, the utilization of the active site was much higher. This proves that the Co_9_S_8_ nanoparticles decorated CNF matrix can effectively store the charges. It should be noted here that Co_3_O_4_/CNF-3 electrode had the lowest specific capacitance of 154 F g^−1^ at 1 A g^−1^. This highlights the importance of the ion-exchange process, which turned the oxide to the more electrochemically active sulfide. Fig. 6(c) shows the specific capacitance of the electrodes measured at different current densities, which reflects the rate capability. Even at 20 A g^−1^, Co_9_S_8_/CNF-3 electrode can still maintain specific capacitance of 292 F g^−1^. Electrothermal impedance spectra were further used to explain the origin of the difference in the electrochemical performance. From Fig. 6(d), all the curves showed straight line in low frequency range, which is the characteristic of capacitive behavior. The curve of Co_9_S_8_/CNF-3 electrodes showed the largest slope, suggesting a lower diffusion resistance. Such results agreed the discussion above that it has the highest capacitance. Overall, the high specific capacitance of Co_9_S_8_/CNF-3 electrode can be attributed to (1) the high loading of the active Co_9_S_8_ phase in the composite, which contributed higher pseudocapacitance and (2) the small size of the Co_9_S_8_ nanoparticles and the highly porous nature of the CNF backbone, which facilitated both the electron and ion transferring in the electrode.

**Fig. 7:**
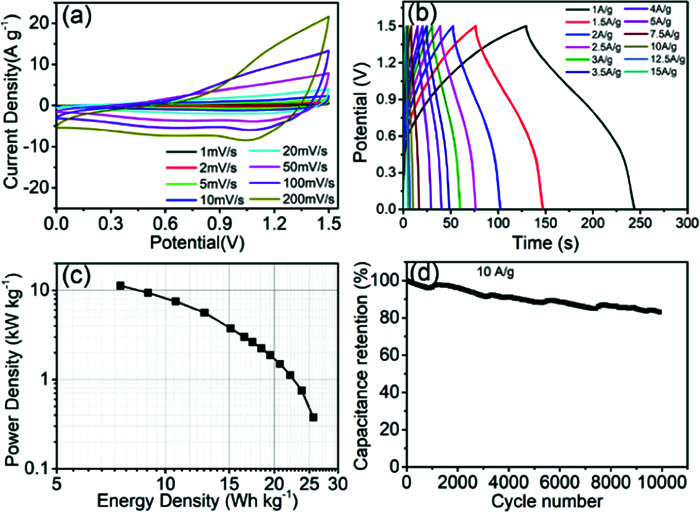
(a) CV curves of Co_9_S_8_/CNF-3//ACNFs capacitor at various scan rates; (b) galvanostatic charge–discharge curves of Co_9_S_8_/CNF-3//ACNFs capacitor at different current densities; (c) Ragone plots of Co_9_S_8_/CNF-3//ACNFs capacitor; and (d) cycling retention curves of Co_9_S_8_/CNF-3//ACNFs capacitor at a current density of 10 A g-1 for 10 000 cycles.

Furthermore, an asymmetric supercapacitor device was assembled by employing Co_9_S_8_/CNF-3 as the positive electrode and activated carbon nanofibers (ACNF) as the negative electrode. Such activated carbon nanofibers were prepared according to previous literature.^26^ The corresponding CV curves at different scan rate can be found in Fig. 7(a). The curve had a near rectangular shape at all scan rates, in the meantime small redox peaks can be also observed. This indicates that both the electrical double layer capacitance (EDLC) and the pseudocapacitance contributed to the charge storage. From CV one can also see that the device can operate at 1.5 V potential window, since no obvious water decomposition current was observed. The galvanotactic charge–discharge curves can be found in Fig. 7(b). The curve had a symmetric quasi-triangle shape, which is the sign of high coulomb efficiency. The specific capacitance of the device was 82 F g^−1^ at 0.5 A g^−1^, and gradually dropped to 48 F g^−1^ at 5 A g^−1^. Even at 10 A g^−1^ current density, the specific capacitance of the device can still reach to 34 F g^−1^, accounting the whole mass of both positive and the negative electrode. The Ragone plot, which reflects the energy and the power density of the device, can be found in Fig. 7(c). The specific energy density was 23.8 W h kg^−1^ when the power density was 0.75 kW kg^−1^. The energy density retained at 10.7 W h kg^−1^ when the power density increased to 7.5 kW kg^−1^. At the highest power density of 15 kW kg^−1^, the energy density can maintain 5.5 W h kg^−1^. These data are superior to some of the cobalt sulfide-based devices, including the hierarchical Co_9_S_8_ nanostructures grown on nickel foam//AC capacitor (21.03 W h kg^−1^ at 0.13 kW kg^−1^ and 13.33 W h kg^−1^ at 1.3 kW kg^−1^) and MOF-derived Co_9_S_8_-carbon//AC capacitor (14.85 W h kg^−1^ at 0.68 kW kg^−1^ and 6.63 W h kg^−1^ at 6.82 kW kg^−1^).^44,45^ The stability of the electrodes was also evaluated by charging the electrode with 10 A g^−1^ current density for more than 10 000 cycles, and the capacitance change was demonstrated in Fig. 7(d). The capacitance retention was 83.1% after the testing, suggesting much better stability compared to those in literature (55–65% retaining after 500 cycles for self-assembled Co_9_S_8_ and 83.3% retaining after 5000 cycles for networked ultralong CoS_1.097_ nanotube).^46,47^

## Conclusions

In summary, Co_9_S_8_ nanoparticles decorated carbon nanofibers composite electrodes were prepared. The porous CNF matrix offers electron and ion transfer pathway, while the Co_9_S_8_ nanoparticles prepared by ion-exchange method owned small size and were uniformly distributed on the CNF surface. Such structure ensured the better utilization of the active Co_9_S_8_ phase to delivery high pseudocapacitance. The optimized Co_9_S_8_/CNF-3 electrode demonstrated high specific capacitance of 718 F g^−1^. Furthermore, high energy density and power density (energy density of 23.8 W h kg^−1^ at the power density of 0.75 kW kg^−1^, energy density of 5.5 W h kg^−1^ at power density of 15 kW kg^−1^) as well as good cycling stability (83.1% performance fading over 10 000 cycles) were demonstrated in the asymmetric capacitor based on such Co_9_S_8_/CNF-3.

## Conflicts of interest

There are no conflicts to declare.

## Supplementary Material

RA-008-C8RA04296A-s001
