# Supplementary material for: Co9S8 nanoparticle-decorated carbon nanofibers as high-performance supercapacitor electrodes
Source: RSC Adv. 2018 Aug 3;8(48):27574–9. doi: 10.1039/c8ra04296a (PMC9086558; doi:10.1039/c8ra04296a)
Supplement: RA-008-C8RA04296A-s001 [file RA-008-C8RA04296A-s001.pdf]

Electronic Supplementary Material (ESI) for RSC Advances.
This journal is © The Royal Society of Chemistry 2018

Co_9S_8 Nanoparticles Incorporated Carbon Nanofibers as High-Performance Supercapacitor Electrodes

Ning Zhang [a], Wencong Wang [a], Changqing Teng [a], Zongxiao Wu [a], Ziran Ye [b], Mingjia Zhi [a*], and Zhanglian Hong [a]

[a] State Key Laboratory of Silicon Material, School of Materials Science and Engineering, Zhejiang University, 38 Zheda Road, Hangzhou, 310027, China

[b] Department of Applied Physics, Zhejiang University of Technology, Zhaohui Campus, Hangzhou, 310014, China

* Corresponding Author: Mingjia_Zhi@zju.edu.cn

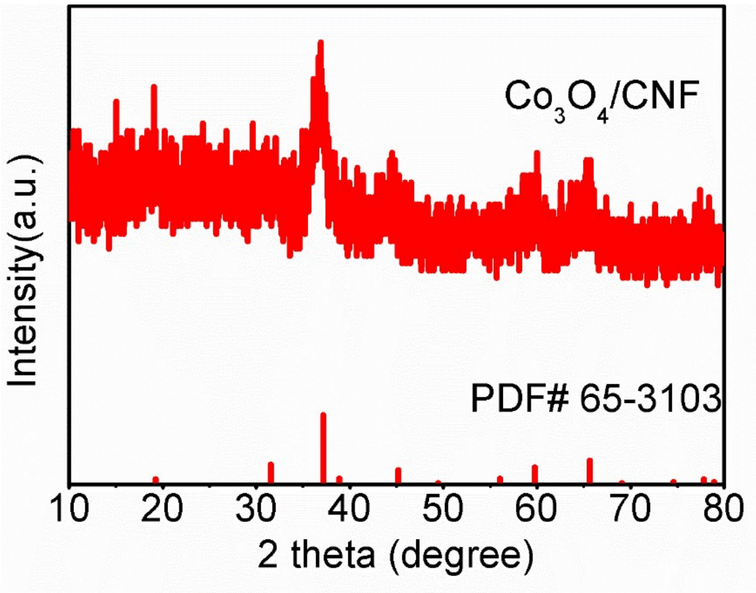

Figure S1: XRD pattern of the Co_3O_4/CNF, the sample was prepared without microwave-assisted sulfurization

Table S1: Elements content of the Co9S8/CNF samples tested by XPS

	Co atomic%	S atomic%	C atomic%
Co_9S_8/CNF-1	5.5	3.9	90.6
Co_9S_8/CNF-2	12.5	10.0	77.4
Co_9S_8/CNF-3	20.5	15.8	63.7

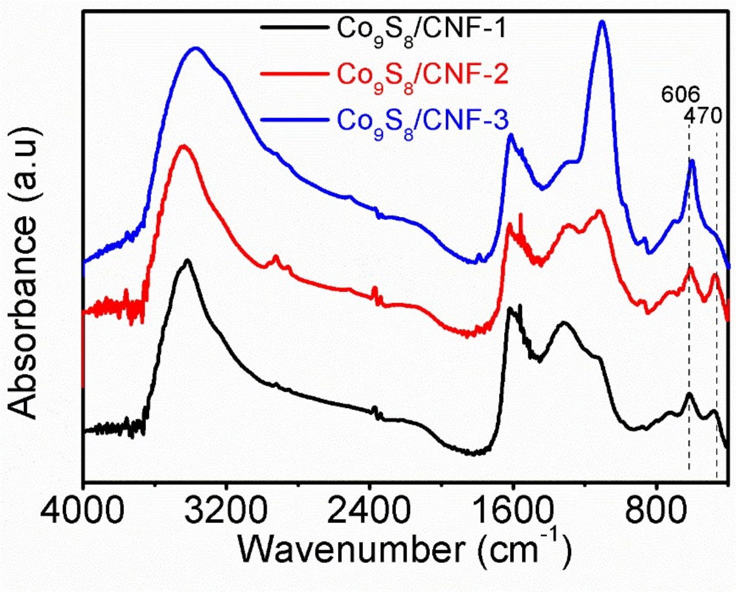

Figure S2: FTIR spectra of the Co_9S_8/CNF samples

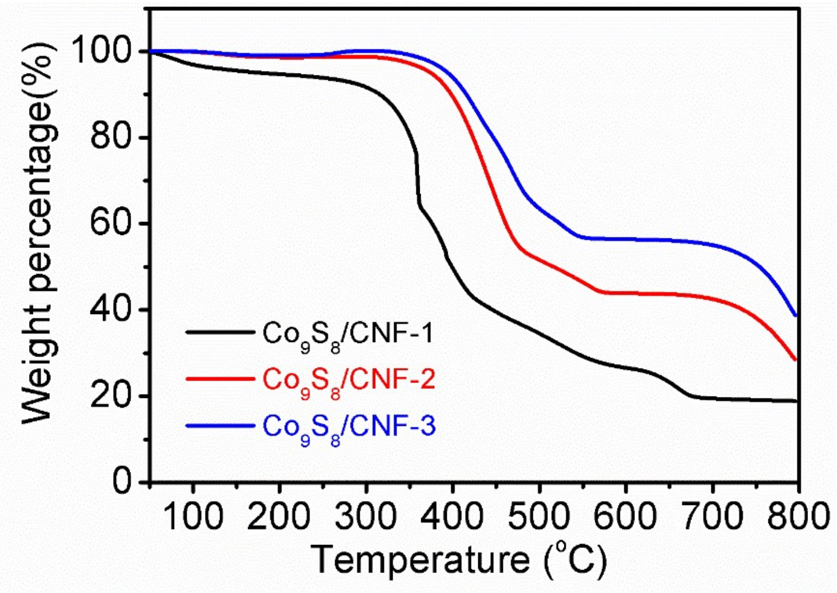

Figure S3: TGA curves of the Co_9S_8/CNF samples

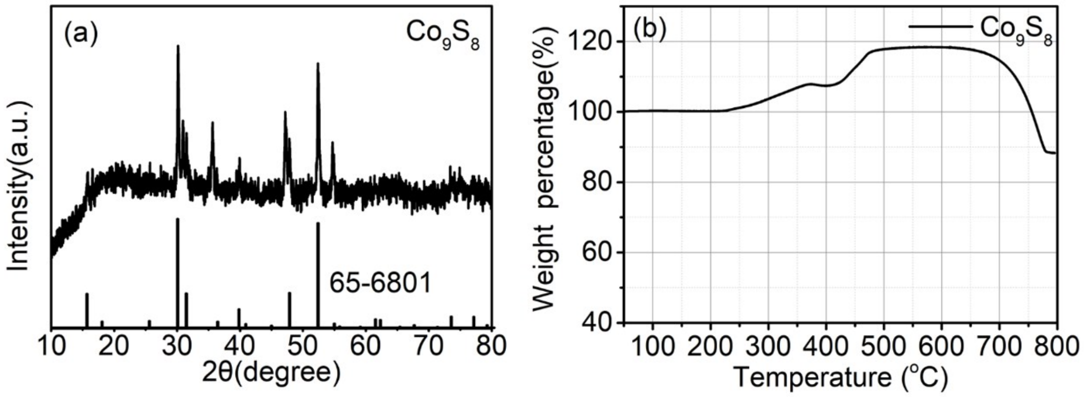

Figure S4: (a) XRD pattern of the Co_9S_8 sample, and (b) TGA curve of the Co_9S_8 sample

The pristine Co_9S_8 was synthesized via a simple one-step microwave hydrothermal process similar to the synthesis of Co_9S_8/CNF series samples. Briefly, cobalt acetates and thioacetamide were dissolved in10 mL deionized water and the concentration was 0.1M. The mixture was then stirred at room temperature until a pink solution was obtained. After that the mixture was loaded in to the microwave hydrothermal apparatus and heated at high temperature. The temperature and the durations were 120 ^{o}C and 1 h. After reaction, the black precipitate was collected, washed with DI water and dried at 60 ^{o}C for 6h.

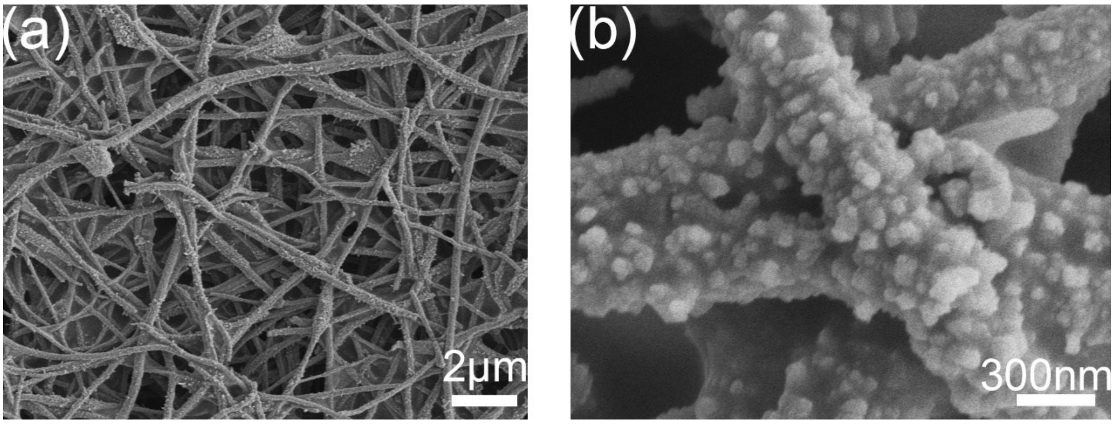

Figure S5: SEM images of the Co_3O_4/CNF-3, (a) low magnification and (b) high magnification

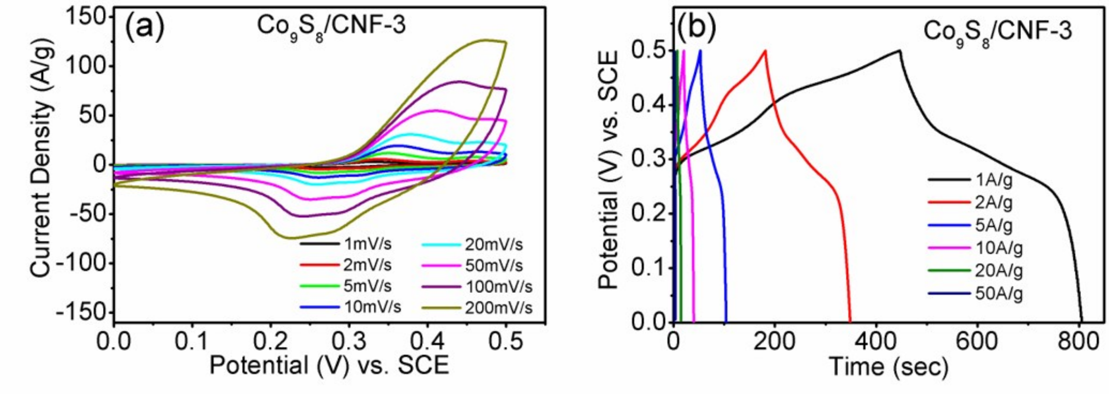

Figure S6: (a) CV curves of Co_9S_8/CNF-3 electrode at different scan rates (1 mV/s to 200 mV/s), and (b) GCD curves of Co_9S_8/CNF-3 electrode at different current densities (1 A/g to 50 A/g)
